# A sinonasal NUT midline carcinoma in an 84‐year‐old man undergoing radiation and proton therapy

**DOI:** 10.1002/ccr3.7262

**Published:** 2023-06-28

**Authors:** Katherynn Zhang, Francisco Laxague, Christina MacMillan, S. Danielle MacNeil, Kevin Fung, John Yoo, Anthony C. Nichols, Adrian Mendez

**Affiliations:** ^1^ Schulich School of Medicine & Dentistry Western University, Lawson Health Research Institute, Western University London Ontario Canada; ^2^ Department of Otolaryngology‐Head and Neck Surgery Western University London Ontario Canada; ^3^ Department of Laboratory Medicine & Pathobiology‐Anatomic Pathology University of Toronto Toronto Ontario Canada; ^4^ Mount Sinai Hospital Sinai Health Toronto Ontario Canada

**Keywords:** ear, nose and throat, oncology

## Abstract

NUT midline carcinomas are rare, aggressive, and poorly differentiated tumors that must be considered in the differential diagnosis of midline head and neck tumors. Despite the scarce data, proton therapy could be an option for some patients.

## Graphical abstract

This is a report of an elderly patient with a NUT midline carcinoma involving the BRD4‐NUTM1 fusion gene, undergoing radiation and proton therapy. NUT midline carcinomas are rare, aggressive, and poorly differentiated tumors that are infrequently reported in literature. To our knowledge, this is the oldest patient reported in the literature, and hopefully, the adopted treatment could be a suitable option in future presentations.

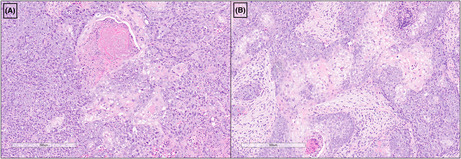

## 1 INTRODUCTION

NUT midline carcinoma (NMC) is a rare tumor of poorly differentiated cells with an aggressive clinical course and a mean survival time of 9.5 months.^1^, ^2^ NMCs present most commonly in the midline structures (head, neck, or mediastinum) but can be associated with other areas.^2^ Genetically, NMCs are characterized by the fusion and rearrangement of the NUT (nuclear protein of the testis) with other genes. The first described NUT‐rearrangement was a translocation between chromosomes 15 and 19.^3^ The t(15;19) translocation results in a BRD4‐NUT oncoprotein, which has been suggested to support the growth of the carcinoma cells by interfering with epithelial differentiation, and accounts for the majority of the NMCs.^4^ Treatment for NMCs usually includes surgery, radiation therapy, chemotherapy, and/or targeted therapy. We aim to present a case of an elderly patient with a NUT midline carcinoma involving the BRD4‐NUTM1 fusion gene, undergoing radiation and proton therapy.

## 2 CASE

**FIGURE 1:**
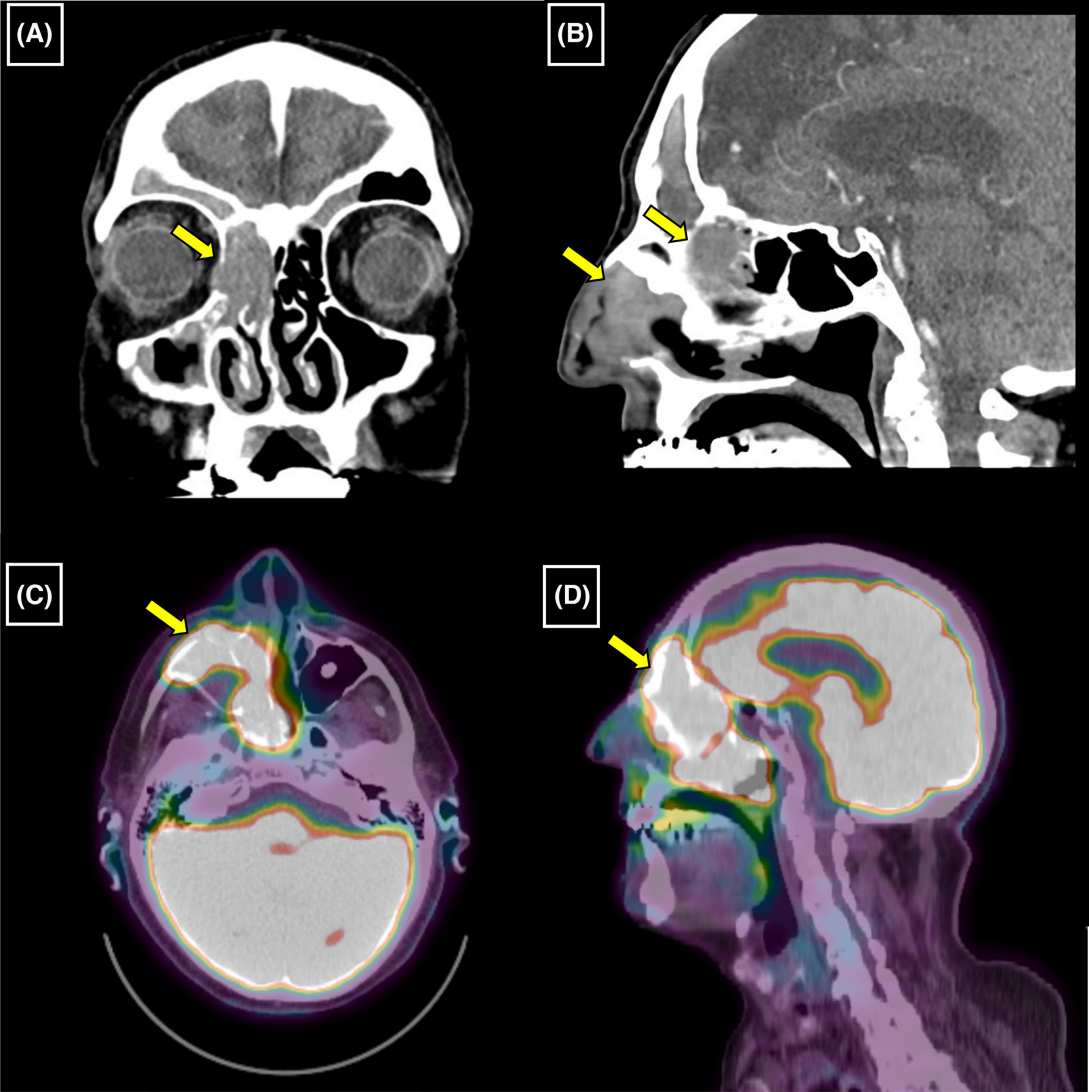
(A, B) Fast growing aggressive mass in the paranasal sinuses greater on the right with focal extension into the right orbit and focal disruption of the right cribriform plate. (C, D) Large intensely hypermetabolic infiltrative mass involving the right paranasal sinuses, right nasal cavity with septal defect and slight extension into the left nasal cavity, medial border of the right orbital cavity, left frontal sinus, right‐sided naso‐ and oropharynx, as described above (SUV max. up to 18.6).

**FIGURE 2:**
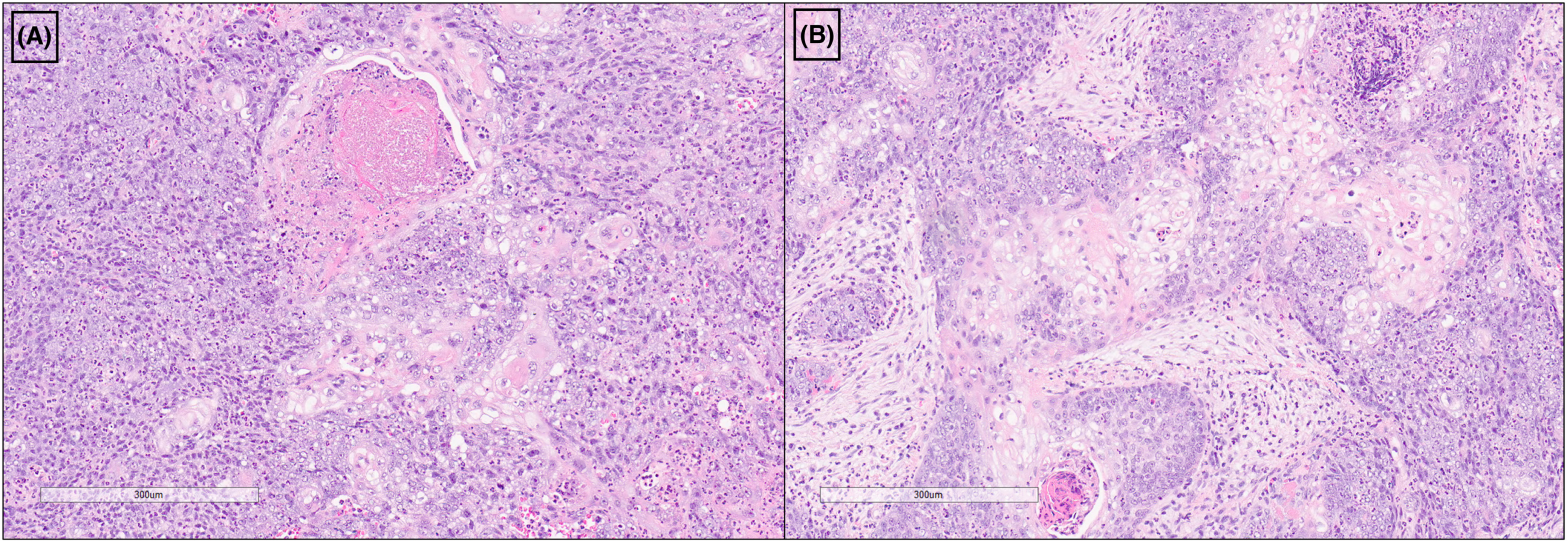
(A, B) Sheets of malignant undifferentiated cells with foci of abrupt keratinization and squamous differentiation. The undifferentiated cells are monotonous and have vesicular to hyperchromatic nuclei with prominent nucleoli. A prominent neutrophilic infiltrate is associated with the tumor.

We present an 84‐year‐old male with a right sinonasal/skull base tumor. He initially presented with progressive right headaches and sinonasal pain, which were examined with imaging and addressed with antibiotic treatment in late 2021. A follow‐up CT‐scan a month after the initial consultation showed a significant change involving an aggressive and infiltrative process in the right anterior ethmoid sinus, right nasal cavity, and left side of the nasal cavity at midline, with medially right orbit invasion (Figure 1A,B). About a week following this presentation, the patient presented at the emergency department and was admitted for two nights due to worsening symptoms of facial and head pain with pressure and epiphora. A PET‐scan was performed showing a large intensely hypermetabolic right paranasal soft tissue mass (SUV max. up to 18.6) involving the ethmoid air cells with erosion of the right lamina papyracea and extending to the medial aspect of the right orbital cavity (Figure 1C,D). The initial clinical diagnosis in February 2022 was an aggressive sinonasal squamous cell carcinoma, including poorly differentiated squamous cell carcinoma, NUT‐midline carcinoma, and DEK‐AFF2‐associated carcinoma among the differential diagnosis. Upon molecular analysis in March 2022, he was confirmed to have NUT‐midline carcinoma based on the presence of a *BRD4* (exon 11 of 20) :: *NUTM1* (exon 2 of 8) gene fusion. Additional studies of biomarkers showed the tumor was negative for EBV by EBER in situ hybridization, had patchy positivity for p16 with 40% positive tumor cells, and was positive for CK5/6, p40, and CD34 immunostains. The tumor cells showed intact staining for INI‐1 immunostain. Microscopic examination of the nasal biopsy showed monotonous sheets of undifferentiated cells with vesicular to hyperchromatic nuclei and prominent nucleoli. Within these sheets, there were discrete foci of squamous cells with keratinization (“abrupt keratinization”). The tumor was infiltrated by numerous neutrophils (Figure 2A,B).

Local treatment options considered for this patient at London Health Sciences Center included cisplatin and docetaxel, radiotherapy with weekly cisplatin, radiation alone, and supportive care. Initially, the patient considered radiotherapy with weekly cisplatin. He ultimately completed a 7‐week course of proton therapy from March to May 2022 in the United States, which was not routinely offered in Canada. He reported mild side effects (skin burns and dryness) managed with over‐the‐counter medications and pain managed with gabapentin (300 mg daily). As of July 2022, it has been 5 months since his NMC diagnosis and over 8 months since his initial presentation, with no evidence of disease at follow‐up post‐treatment.

## 3 DISCUSSION

In the literature, a total of 27 studies exist with information about 106 patients with NMCs. The data represented 59/106 (55.7%) female patients with a mean age of 36.7 at the time of diagnosis, ranging from 8 to 78 years. The most common tumor sites include primary tumors in the parotid glands and sinonasal cavity/tract, with swelling and pain around those sites as the initial symptoms. Treatment methods for NMCs include surgery, radiation therapy, chemotherapy, and/or targeted therapy. Their mean survival time after initial diagnosis is 7.3 months (range: 1–22 months). Out of the treatment particulars described for some cases, patients usually undergo a multimodal approach including surgery in combination with (neoadjuvant or adjuvant) chemotherapy and/or radiation therapy (*n* = 53, 1 with investigational target therapy), surgery only (*n* = 1), chemotherapy and/or radiation therapy with no surgery (*n* = 17, 6 of which also had targeted therapy). A small number undergo palliative care (*n* = 3) or no treatment (*n* = 1). Currently there is no established consensus on a standard treatment guideline for NMCs.^2^ This case is consistent in initial presentation and tumor location with other reported head and neck NMC cases; however, the patient's age is much older than previous reported cases. He pursued proton therapy as his treatment choice, which, to our knowledge, had not been mentioned in previous reports. Beams of proton therapy have a high precision for the target tumor with minimized excess energy that could damage surrounding normal tissue, which makes the therapy suitable for pediatric cancers, head and neck tumors, and brain tumors. Proton therapy has been demonstrated to have clinical benefits and cost‐effective potential. However, the treatment is not widely available and not routinely offered in many countries.^5^

## 4 CONCLUSION

NUT midline carcinomas are rare, aggressive, and poorly differentiated tumors that must be considered in the differential diagnosis of midline head and neck tumors. Scarce information exists regarding the best treatment modality, specially for elderly patients due to the low number of reports in the literature. To our knowledge, this is the oldest patient reported in the literature, and hopefully, the adopted treatment could be a suitable option in future presentations.

## CONSENT STATEMENT

Written informed consent was obtained from the patient to publish this report in accordance with the journal's patient consent policy.

## AUTHOR CONTRIBUTIONS

**Katherynn Zhang:** Data curation; formal analysis; methodology; project administration; writing – original draft; writing – review and editing. **Francisco Laxague:** Data curation; formal analysis; investigation; methodology; writing – review and editing. **Christina MacMillan:** Data curation; formal analysis; writing – review and editing. **stephanie danielle mac neil:** Resources; supervision; validation. **Kevin Fung:** Resources; supervision; validation. **John Yoo:** Resources; supervision; validation. **Anthony C. Nichols:** Resources; supervision; validation. **Adrian Ivar Mendez:** Conceptualization; investigation; methodology; resources; supervision; validation; writing – original draft; writing – review and editing.

## FUNDING INFORMATION

The Institutional Review Board/Research Ethics Board of our institution approved this study. Patient consent was obtained and included with the submission of this manuscript.

## CONFLICT OF INTEREST STATEMENT

The authors have no conflicts of interest or financial disclosures to declare.

## Data Availability

The data that support the findings of this study are available from the corresponding author upon reasonable request.
